# Efficacy of combined orthokeratology and 0.01% atropine for myopia control: the study protocol for a randomized, controlled, double-blind, and multicenter trial

**DOI:** 10.1186/s13063-021-05825-1

**Published:** 2021-12-01

**Authors:** Ying Yuan, Chengcheng Zhu, Mingming Liu, Yali Zhou, Xiao Yang, Bingru Zheng, Zhouyue Li, Xinjie Mao, Bilian Ke

**Affiliations:** 1 Department of Ophthalmology, Shanghai General Hospital, Shanghai Jiao Tong University School of Medicine, Shanghai, 200080 China; 2 National Clinical Research Center for Eye Diseases, Shanghai, China; 3 Shanghai Key Laboratory of Fundus Disease, Shanghai, China; 4 Shanghai Engineering Center for Visual Science and Photomedicine, Shanghai, China; 5 Shanghai Engineering Center for Precise Diagnosis and Treatment of Eye Diseases, Shanghai, China; 6 State Key Laboratory of Ophthalmology, Zhongshan Ophthalmic Center Sun Yat-Sen University, Guangzhou, China; 7 School of Ophthalmology and Optometry and Eye Hospital, Wenzhou Medical University, 270 Xueyuan Road, Wenzhou, 325027 Zhejiang China

**Keywords:** Myopia control, Orthokeratology, 0.01% atropine, Pediatrics, Multicenter trial

## Abstract

**Background:**

The prevalence of myopia is increasing worldwide and is presently recognized as a major public health issue. Researchers and clinicians have been devoted in exploring appropriate clinical interventions to slow its progression in children. Mounting publications have proven that both orthokeratology (OK lens) and 0.01% atropine eyedrop can retard eye growth and myopia progression. However, it remains unclear whether the combination of OK lens and 0.01% atropine has the potential to magnify the effectiveness of myopia control. The present study aims to compare the myopia control efficiency of the combination of OK lens and 0.01% atropine with the monotherapy of OK lens in children.

**Methods:**

The present study is a randomized, controlled, double-blind and multicenter clinical trial. A total of 96 children within 8–12 years old were recruited. These participants are treated with the combination of OK lens and 0.01% atropine eyedrop or the combination of OK lens and placebo eyedrop. Each group includes 48 participants. The inclusion criteria are as follows: myopia between − 1.00 and − 4.00 D in either eye and astigmatism of no more than 1.50 D. The follow-up time points will be 1, 6, 12, 18, and 24 months from randomization. The primary outcome is determined by the difference in axial length of the two groups, between the baseline and 24 months from randomization.

**Discussion:**

The present randomized, controlled clinical trial would indicate the additive effects of the combination of OK lens and 0.01% atropine, and the extent of these effects, in retarding myopia progression and axial elongation in children.

**Trial registration:**

Chinese Clinical Trial Registry (ChiCTR), ChiCTR1800018419. Registered on 17 September 2018. http://www.chictr.org.cn/showproj.aspx?proj=29216

**Supplementary Information:**

The online version contains supplementary material available at 10.1186/s13063-021-05825-1.

## Background

The prevalence of myopia is dramatically increasing, worldwide, especially in East Asia [1]. Recent studies have indicated that the prevalence of myopia in East Asian countries has reached as high as 90% [2]. It is estimated that there will be approximately five billion people (half of the world’s population) with myopia by 2050 [1]. In addition, myopia occurs at younger ages. A number of studies have presented that children who develop myopia at a younger age is prone to progress more quickly and ultimately progress to high myopia [3, 4]. Myopia progression is usually accompanied by the elongation of the axial length, which may result in vision-threatening pathology, such as macular degeneration, retinal detachment, and glaucoma [5, 6]. Given the high prevalence of myopia and its strong association with sight-threatening ocular diseases, myopia has become a major public health concern. Hence, it is critical to explore effective strategies to control myopia progression.

At present, numerous studies have assessed the efficacy of OK lens and atropine for myopia control in children [7–9]. OK lens was originally designed to correct myopia and provide clear unaided visual acuity during the day. At present, increasing evidence has shown that OK lens is effective for slowing axial elongation and myopia progression. A single-masked randomized clinical trial conducted in Hong Kong revealed that the increase in axial length in the OK lens group was less than that in the single-vision spectacle glasses group (0.36 mm vs. 0.63 mm) at 2 years [7]. In the cohort study conducted in Japan, the increase in axial length for over 2 years was 0.39 mm and 0.61 mm in the OK lens and the single-vision spectacle glasses groups, respectively [8]. In a retrospective cohort study conducted in China, the OK lens group exhibited less changes in axial length, when compared to the single-vision spectacle glasses group, at 2 years (0.34 mm vs. 0.70 mm) [9]. In conclusion, OK lens can induce a 40–60% mean reduction in axial elongation, when compared to single-vision spectacles, in terms of correcting myopia, at present.

Other than OK lens, topical atropine therapy has also been demonstrated to be effective in retarding myopia progression in children. High-dose atropine (1% and 0.5%) and moderate-dose atropine (0.1%) have been shown to have a dose-dependent retardation effect [10, 11]. However, these doses usually induced photophobia side-effects during treatment, which resulted in a high dropout rate [11, 12]. In addition, the rebound effect after atropine discontinuation has been reported, and this was especially notable in higher concentrations of atropine [13]. Recently, several studies have reported that 0.01% atropine is effective for slowing myopia progression, with negligible side-effects [14, 15]. The 5-year study of atropine treatment for myopia (ATOM2) revealed that for the myopia progression of 0.01% atropine, in terms of refraction, this was found to have a similar efficacy as that of 0.5% and 0.1% atropine at the end of 2 years [16]. After the “wash-out period” of the third year, the overall progression of myopia in the 0.01% atropine group was significantly lower than that of the 0.5% and 0.1% atropine groups. Then, the study continued to restart the 0.01% atropine for two further years. At the end of 5 years, the overall progression of spherical equivalence myopia in the 0.01% atropine group was significantly lower, when compared to the 0.1% and 0.5% atropine groups. Despite the encouraging overall results of the ATOM2 study, not all participants responded well to atropine. In the ATOM2 study, 9.3% of children in the 0.01% group had myopia progression of ≥ − 1.5 D over the initial 2-year treatment. This was higher than that in the 0.5% and 0.1% groups. However, in the LAMP study, the use of 0.01% atropine had a minimal effect in controlling the myopia progression and axial elongation over 1 year [17]. In addition, spectacle lenses or contact lenses are needed during the daytime, since atropine solutions cannot correct the myopia.

The exact mechanism of the myopia control of OK lens or atropine eyedrop is presently not fully understood. Recently, OK lens is known to control myopia progression by reducing the peripheral hyperopic defocus [18]. Atropine is thought to have effects on anti-muscarinic receptors of the retina and sclera [19]. Chen et al. reported that large pupil diameters can facilitate the effect of OK lens to slow axial growth in myopia [20]. In addition, 0.01% atropine eyedrop were proven to have a significant increase in pupil size [21]. Thus, the combination of OK lens and 0.01% atropine treatment may have great potential in magnifying the effectiveness of myopia control. In a previous randomized clinical trial conducted in Japan, the combination therapy was more effective in slowing axial elongation than monotherapy of OK lens over the 2 years (0.29 mm vs. 0.40 mm) [22]. Pauline and his colleagues set a randomized, observer-masked, prospective study in Hong Kong [23]. They found the additional use of 0.01% atropine did not affect the OK lens performance or clinical responses after 1 month of treatment [23]. The possible synergistic effect of combination treatment for myopia control needs longer study duration. However, these studies were not masked to the participants, and the control group only receives OK lens treatment without placebo eyedrop. Thus, we conducted a randomized, controlled, double-blind and multicenter trial to investigate the additive effects of the combination of OK lens and 0.01% atropine eyedrop in slowing axial elongation in children with myopia.

## Methods/design

**Fig. 1:**
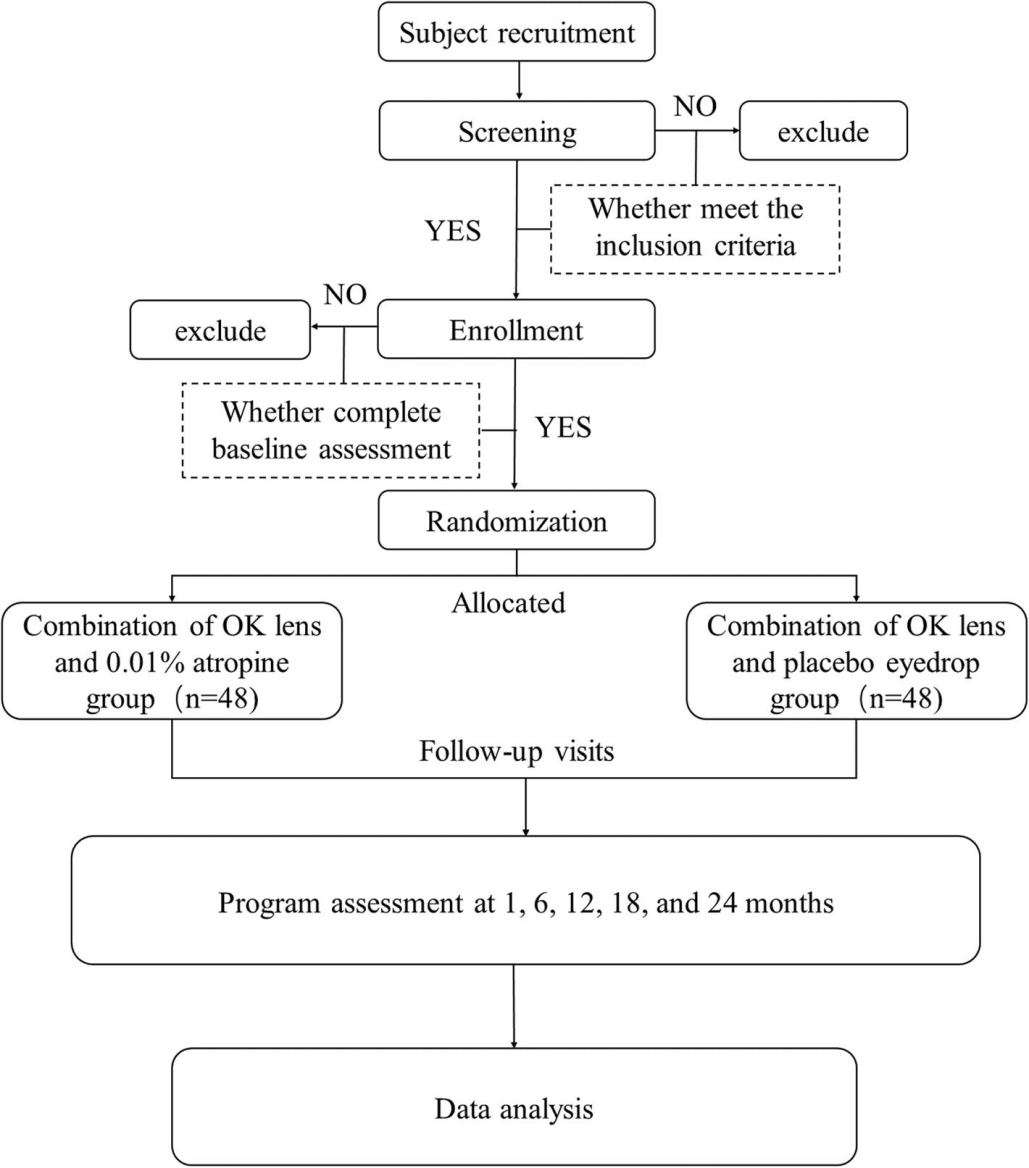
Study flow diagram

The present prospective, multicenter, randomized controlled, double-blind clinical trial aimed to evaluate the efficacy of the combination of OK lens and 0.01% atropine for the treatment of childhood myopia. The clinical trial is presently being undertaken in Shanghai General Hospital, Wenzhou Medical University, and Zhongshan Ophthalmic Center, Sun Yat-sen University, China. The trial is being coordinated by Shanghai General Hospital. Ethical approval was obtained from the Institutional Review Board of Shanghai General Hospital, Wenzhou Medical University, and Zhongshan Ophthalmic Center, Sun Yat-sen University. Written informed consent was collected from the participants and their parents/guardians prior to the enrolment. The schedule of enrolment, intervention, data collection, and assessment conform to the Standardized Protocol Items: Recommendations for Interventional Trials (SPIRIT) guidelines (Table 1, Additional file 1). The study flow schedule is shown in Fig. 1.

**Table 1:**
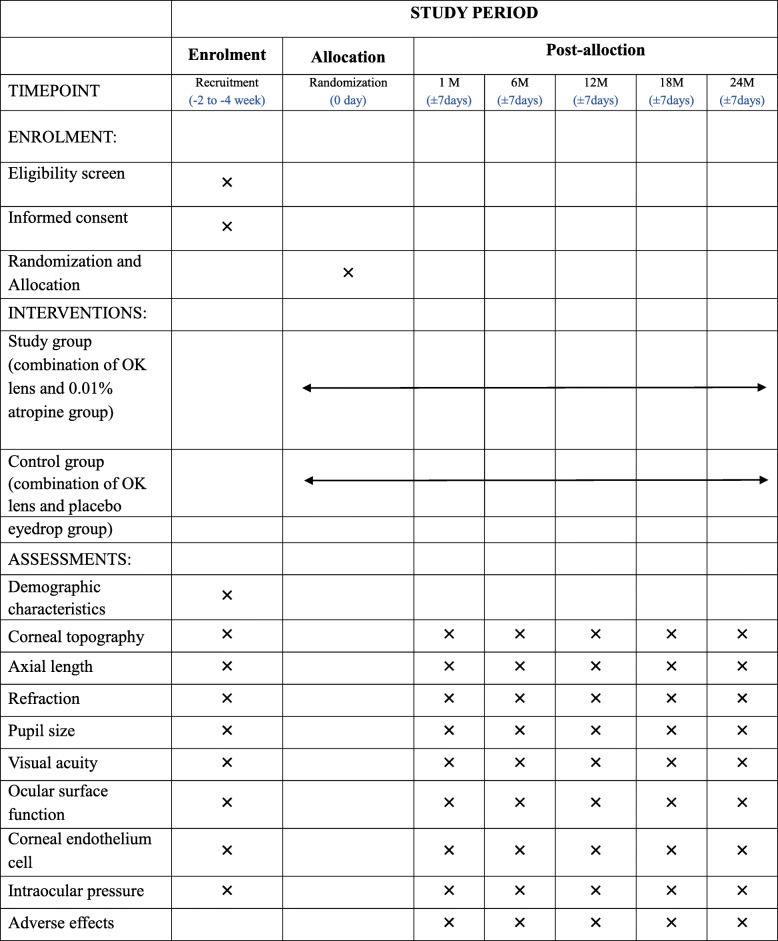
Standard Protocol Items: Recommendations for Interventional Trials (SPIRIT)

### Participant eligibility

#### Inclusion criteria

The participants were within 8–12 years old. These participants intended to undergo the OK lens treatment. The inclusion criteria for the present study were as follows: spherical equivalent refractive error between − 1.00 and − 4.00 D in either eye, astigmatism of no more than 1.50 D in either eye, a best corrected visual acuity (BCVA) of no worse than 25/25 in both eyes, the weight of birth was no less than 1500 g, the patient/guardian agrees to be randomized, and attends the scheduled and follow-up visits.

#### Exclusion criteria

The exclusion criteria were as follows: patients with ocular disorders, such as strabismus, amblyopia, cataract, or ptosis; patients previously used OK lens or atropine eyedrop to prevent myopia progression; patients with disorders contraindicated to atropine, such as known allergies, cardiovascular disease, or epilepsy; patients with disorders contraindicated to OK lens wear, such as ocular inflammation or infection; and patients with systemic disorders that might affect refractive development, such as Down syndrome or Marfan’s syndrome.

### Randomization and masking

A statistician was responsible for the randomization of the 0.01% atropine eyedrop or placebo eyedrop, which was achieved using a web-based randomization program. The randomization was stratified by center with fixed block size of 4. Cards that identified the combination of OK lens and 0.01% atropine group and OK lens group were placed in sealed opaque envelopes and kept by the principal investigator until the conclusion of the trial. The principal investigator should open the opaque envelopes in an emergency.

The 0.01% atropine eyedrop and placebo eyedrop were prepared in a manner that appeared similar in appearance. The manufacturer of the eyedrop labeled the investigational eyedrop according to the randomization code number. Then, the manufacturer delivered the pre-randomized eyedrop to each recruitment center. The masked investigator was responsible for the ophthalmic measurements but was not allowed to handle the eyedrops throughout the study. During the clinical trials, neither the participants nor the masked investigator were aware of the group allocation. The masked clinical research coordinator (CRC) guided the participants in performing and finishing each of the examinations. All outcome assessments were performed by masked investigator.

### Recruitment

Participants were recruited from outpatients at each study site. The potentially eligible participants and their parents or guardians were contacted by CRC. And the CRC introduced the study to the participants and their parents or guardians in detail. The interested participants and their parents or guardian were invited to perform a screening examination to assess their eligibility. At the enrolment visit, the participants and their parents or guardian signed the informed consent before the examination.

### Intervention

Eligible participants were randomly assigned to the combination of OK lens and 0.01% atropine group, or the combination of OK lens and placebo eyedrop group, at a 1:1 ratio. Participants were recruited from 3 hospitals in China. To ensure balance in study site, a stratified and block randomization algorithm was used and randomization was stratified by study site and block sizes of four were used within each strata. The 0.01% atropine and placebo eyedrop looked identical in the appearance and was labeled according to the randomization code number. The participants got the eyedrop that corresponded to the assigned consecutive number in accordance with their entrance to the study.

The treatment for the combination of OK lens and 0.01% atropine group comprised of the instillation of one drop of 0.01% atropine in each eye, at 10 min before OK lens insertion, and at least 8 h of overnight wear of OK lens. Participants in the OK lens group wore the OK lens every night after the placebo eyedrop instillation. Under the condition of illness or the presence of any abnormal ocular symptoms, the nightly wear of the OK lens and use of the atropine or placebo eyedrop would both be suspended. Follow-up visits were performed at 1, 6, 12, 18, and 24 months from randomization. We provided the 3-month dosage of 0.01% atropine eyedrop or placebo eyedrop at a time. The parents or guardians are required to return the consumed vials to the investigator every 3 months. The eyedrops were being sent to minimize dropouts during the pandemics.

### Primary outcome

The primary outcome was to determine whether the combination of OK lens and 0.01% atropine intervention are superior to OK lens, in terms of preventing myopia progression, by evaluating the axial length elongation in these two groups of participants. For the primary outcome analysis, the myopia progression over 2 years would be determined by the difference in axial length between the baseline and 24-month visit. Other measurements obtained at the follow-up visits were considered as secondary outcome measures.

### Secondary outcome

The secondary outcome was the comparison of the elongation of axial length between the combination of OK lens and 0.01% atropine group and OK lens group after 1 year. The change of pupil size and refraction between the baseline and 24-month visit will also be calculated as the secondary outcome. In addition, the safety was evaluated through the corneal endothelia cell and ocular surface function in these two groups of participants at each follow-up visit.

### Sample size calculation

The two-sample *t* test for superior statistical analysis was used for the sample size calculation. The parameters used for the sample size calculation included the following: a significance level of 0.05, 95% confidence interval, 80% power, and 1:1 allocation. According to previously published articles, the axial length elongation in the combination of OK lens and 0.01% atropine group was preset at 0.09 ± 0.12 mm, while the OK lens was preset at 0.19 ± 0.15 mm in 1 year [24]. According to the previous experience of the investigators with clinical trials for treating myopia and other published data, the rate of participant loss to follow-up over 2 years was estimated to be 20%. Calculations, which were two-sided, were performed using the software, NCSS PASS 11. A total sample of 96 eligible children was required in the trial, with 32 participants being planned to be assigned to each of the three study sites.

### Data collection, management, and monitoring

Before the start of the trial, all investigators from each site have been trained in the clinical trial protocol, data management, and methodology of indicator evaluation. Electronic case report form (CRF) was designed by the statistician according to the trial protocol and medical record. Participant log was distributed to each patient to record their daily use of OK lens and eyedrops. During the study, investigators collected the data and recorded it on the participant medical record.

In the present study, the main adverse events were mainly conjunctivitis or keratitis after wearing OK lens and sensitivity to light or ocular irritation after instillation of eyedrops. The masked investigators examined cornea and conjunctiva using the slip-lamp microscopy at each visit time. Once the presence of any abnormal ocular symptoms occurs, the nightly wear of the OK lens and use of the atropine or placebo eyedrop would both be suspended. And at least one masked investigator would review and report the signs and symptoms in a timely manner according to the procedures. Each adverse event would be recorded in the CRF. Participants who are unable to be followed up throughout the study are considered dropout of our study. Once the intolerable side effects occur or the effect of treatment is poor, the participants could withdraw from the study. Additionally, participants could withdraw from the study for any reason at any time. The investigators may also withdraw participants from the study to protect their safety.

To ensure the quality of the clinical trial, an independent committee, including statisticians, ophthalmologist, and members of the ethics committee of Shanghai General Hospital, was established and oversaw the study throughout the study period. They monitored the progress and safety of the study and reported their recommendation to the principal investigator. This committee was independent from the trial researchers. Participant medical records were stored securely in locked cabinets in the research laboratory at each site. Only the investigators and monitors have access to participant medical records. The data and information of the participants would not be used in other ancillary studies. In order to protect the privacy of the participants during data analysis, their identity information was hidden. There was no interim analysis and no stopping rules in this study. Auditing was performed by Good Clinical Practice (GCP) office of Shanghai General Hospital. They reviewed the progress of the trial every six months. The process was independent of investigators.

### Statistics analysis

The patients who took eyedrops at least once after randomization and have corresponding efficacy evaluation were analyzed as the full analysis set (FAS). In the primary analysis, missing values in FAS will not be imputed, but multiple imputation will be used to deal with missing data in sensitivity analysis. Robustness of the results will be evaluated through sensitivity analysis in the per protocol set (PPS). And the PPS refers to subjects who were able to wear OK lens and eyedrops on schedule for 2 years. Patients who received study treatment at least once with safety assessment during the trial were analyzed as safety set (SS). The primary outcome was analyzed in the FAS and PPS, and secondary outcomes were analyzed only in the FAS. The safety was evaluated in the SS.

Continuous data were presented as the mean ± standard deviation (SD) or medium (P25-P75), while categorical data were presented as numbers (percentages). For the primary outcome, ANCOVA model with center effect correction was used to compare the difference of change from baseline between two groups and calculated its 95% CI. For the secondary efficacy outcomes, ANCOVA models were used to compare the difference of change from baseline between two groups. The distribution of adverse events of the two groups is described, and chi-square test or Fisher’s exact probability test was employed for comparison of incidence between the two groups. There was no predefined subgroup analysis in this study. All statistical analyses were performed with SAS software (version 9.4), and statistical significance was set as *P* < 0.05 with a two-sided test unless otherwise noted.

### Post-trial care

It is recommended that children with myopia should be re-examination at least half a year, to identify whether spectacles or OK lens need to be replaced. They can continue examining visual acuity, axial length, refraction, and so on in our outpatient clinics.

### Protocol amendments

Any protocol modifications that may impact on the conduct of the study including changes of study objectives, study design, sample sizes, or study procedures required a formal amendment to the protocol. These amendments were approved by the institutional review board prior to implementation. Minor protocol changes that have no effect on the study were agreed by the principal investigator and were documented in a memorandum.

## Discussion

The present trial was designed to investigate the efficacy of the combination treatment of OK lens and 0.01% atropine. According to previous studies and the understanding of the mechanism of myopia progression, the additive effects of retarding myopia progression through the combination of OK lens and atropine may be obtained. To the best of our knowledge, the present study is the first randomized, controlled, double-blind, and multicenter trial that compared the efficacy of the combination of OK lens and 0.01% atropine with the combination of OK lens and placebo eyedrop.

It has been widely accepted that the underlying mechanism of retarding the myopia progression of OK lens was inducing myopic defocus [25]. A clinical study revealed that the choroidal thickness after OK lens treatment increased, when compared to that in the wearing single-vision spectacle glasses group [26]. Recently, it was reported that 0.01% atropine could also significantly increase the choroidal thickness of the eyes of young myopic children [27]. Samuel T.-H. Chiang et al. reported that myopic defocus can induce additional choroidal thickening in children, whose choroidal thickness has already been increased by the nightly treatment with atropine eyedrops [28]. The combination of OK lens and atropine induced a greater increase in choroidal thickness, when compared to the monotherapy with OK lens or atropine [29]. Therefore, the clinical evidence indicates the additive effect of the combination of OK lens and atropine for myopia control [23, 24, 30, 31].

In conclusion, the combination of OK lens and 0.01% atropine eyedrop has gained great interest in the retardation of myopia progression. The present study aimed to investigate the myopia control efficacy of the combination of OK lens and 0.01% atropine over 2 years of follow-up in 8–12 year-old school children. These results would broaden the understanding of whether the combination of OK lens and 0.01% atropine can retard myopia progression and its extent.

## Trial status

At the time of submission, recruitment has already ended, and all the participants were under follow-up visits. The recruitment date of the first patient is 25 January 2019. The date of recruitment of the last patient is 13 July 2020. A total of 96 children were recruited. The 24-month visit is expected to be completed on 17 August 2022. The work was delayed due to the coronavirus pandemic. The protocol version is 1.0, dated 20 April 2018.

## Supplementary Information

**Additional file 1.**

**Additional file 2.**

**Additional file 3.**

## Data Availability

There is no plan for public access to the dataset of this trial now. The Institutional Review Board, auditing and monitoring committee can get access to the dataset during the study. The principal investigator will supervise the management of the final trial dataset with the statistician. Personal identification information will be hidden in the final trial dataset. The study dataset used or analyzed are available from the principal investigator on reasonable request after the trial is completed. We have clarified in the manuscript.

## Abbreviations

ChiCTR: Chinese Clinical Trial Registry
OK lens: Orthokeratology
BCVA: Best corrected visual acuity
CRC: Clinical research coordinator
CRF: Case report form
GCP: Good clinical practice
FAS: Full analysis set
LOCF: Last-observation-carried-forward
PPS: Per protocol set
SS: Safety set
SD: Standard deviation
IRB: Institutional review board
