# Supplementary material for: Efficacy of combined orthokeratology and 0.01% atropine for myopia control: the study protocol for a randomized, controlled, double-blind, and multicenter trial
Source: Trials. 2021 Dec 1;22:863. doi: 10.1186/s13063-021-05825-1 (PMC8633914; doi:10.1186/s13063-021-05825-1)
Supplement: Supplementary file 2 — Additional file 2. [file 13063_2021_5825_MOESM2_ESM.doc]

| **筛选号**： |  |
| --- | --- |

比较角膜塑形技术联合0.01%阿托品滴眼液与单用角膜塑形技术两种方式控制儿童近视进展的随机、对照、双盲、多中心临床研究

**（临床验证）**

|  |  |
| --- | --- |

**知 情 同 意 书**

**（8岁-12岁专用版，第1.1版，2018年11月08日）**

| 受试者姓名： | ____________________________ |
| --- | --- |
| 联系地址： | ____________________________ |
| 联系电话： | ____________________________ |
| 研究中心名称： | ____________________________ |
| 研究中心编号： | ____________________________ |
| 研究者： | ____________________________ |

**知情同意书**

**研究标题：**比较角膜塑形技术联合0.01%阿托品滴眼液与单用角膜塑形技术两种方式控制儿童近视进展的随机、对照、双盲、多中心临床研究

**研究目的：**评价角膜塑形技术联合0.01%阿托品滴眼液与单用角膜塑形技术两种矫正方式控制儿童近视进展的安全性及有效性

**受试者须知**

你的医生想要了解一种角膜塑形技术联合0.01%阿托品滴眼液是否安全以及是否能帮助和你一样患有近视的儿童，想邀请你参加这项临床研究。在决定是否参加这项研究前，你和你的监护人需要了解我们为什么要进行这项研究以及它将涉及什么。你可以与你的朋友、亲属和医生一起仔细阅读和讨论以下资料。有任何不清楚的地方或想了解更多信息，现在或在研究过程中任何时候有任何问题，研究人员都可以随时解答。请仔细考虑后再做决定，如果参加此项研究，你和你的监护人均须在此知情同意书上签名。

若你正在参加其他的研究，请告知你的研究医生或者研究人员。

**本项研究的目的**

本项研究目的是评价角膜塑形技术联合0.01%阿托品滴眼液与角膜塑形技术两种矫正方式控制儿童近视进展的安全性及有效性。这项研究将有3个研究中心96名受试者参加。你的研究医生会告诉你研究人数是否已经达到总数，以及是否需要你参加研究。也有可能在筛查期中获得的资料说明你不适合参加此临床研究。

**如果参加研究，需要做些什么**

如果你和你的监护人同意参加研究，签署了知情同意书，你的研究医生将对你进行检查，以确定你是否符合要求参加此项研究。

首次访视（筛查访视）时研究人员或医生会询问你现在或既往的医疗情况，以及曾经服用或正在服用的可能影响视力的药物或补充剂，记录你的年龄、身高、体重等一般资料，检查你的屈光情况。若这些检查结果适合此项研究，医生将要求你做更详细的验光、眼轴、眼压、角膜地形图、角膜内皮计数、角膜荧光素染色、泪河高度、泪膜破裂时间检查。

如果医生确定你符合所有条件并可以参加此项研究，你将被随机分配到角膜塑形技术联合0.01%阿托品滴眼液组或角膜塑形技术组中的任意一组，连续治疗两年。同时，医生将依据上述检查结果，为你验光配角膜塑形镜进行屈光矫正或介绍0.01%阿托品滴眼液的使用方法。期间你至少要来医院就诊7次，（包括筛选期，取OK镜及阿托品滴眼液（治疗开始）、治疗后1个月、6个月、12个月、18个月、24个月随访）。每次研究访视将询问产品使用情随访况、治疗感受，并安排进行详细的视功能及眼部生物学参数检查，包括验光、眼轴、眼压、角膜地形图、角膜内皮计数、角膜荧光素染色、泪河高度、泪膜破裂时间。

研究人员会为你安排好所有访视，并确保你和你的监护人知道何时来访视。每次访视需携带研究产品。

研究期间允许服用的药物有些限制，研究医生会向你解释。若其他医生给你使用药物或医疗器械，需尽快与你的医生沟通，以免因交叉影响干扰试验结果。

研究中你的任何感受记得告诉你的监护人和医生，如果感到不适或服用任何药物时，必须告诉你的研究医生或监护人，这些信息对本项研究很重要。你或你的监护人可在任何时候给研究医生联系。

**参加本研究可能的风险**

与任何其他医疗器械一样，部分受试者配戴角膜塑形镜后可能会有不适，如频繁眨眼、视疲劳和干眼等。研究过程中，你可能会出现或者不发生上述不适。你和你的监护人应该与研究医生一起讨论。

0.01%阿托品使用的部分受试者，可能会出现畏光、瞳孔放大效应。研究医生将密切关注你病情波动的迹象。如果出现任何不适，不论其是否与治疗有关，请务必立即告知研究医生，以获得帮助和及时处理。

除了上述已知可能的风险，研究产品或研究本身还可能存在一些无法预知的风险、不适。如果发现新信息，研究医生会及时通知你，必要时补充知情同意书的内容，重新获得你和你的监护人的知情同意。

**参加本研究对你的帮助**

参加本项研究，研究人员会安排支付你由于参加研究造成的相关费用（例如与本研究相关的各项检查费及试验药品费用）。

参加本研究你的近视进展可能会得到控制，但并不保证一定直接受益。

通过参加本研究，你可能会帮助到患有和你相同疾病的小朋友。

**研究中你享有的权益**

参加本研究完全自愿，并且你和你的监护人可以选择任何时候停止参与。如果决定退出研究，请联系研究医生，他们会向你解释停止参加研究的最佳方法。

此外，你也可能因为以下任何一个原因被要求退出研究：没有遵从研究医生的指示；没有正确使用产品；如果研究医生认为退出研究对你的健康和利益而言是最佳选择；如果研究没有足够的受试者或已经达到了所需数量的受试者。无论你决定不参加研究还是退出研究，都不会对你现在的或将来的医疗服务造成负面影响。即使你签署这份知情同意书，你仍保留所有的合法权利。

如果你在筛选和入组时仅单眼符合入组标准，则该眼为研究眼，在治疗期间如果你非研究眼出现近视需要干预治疗，研究者会根据你近视的情况选择相应的治疗方案（包括免费提供角膜塑形镜或阿托品滴眼液），直至研究结束。如果在筛选和入组时双眼符合入组标准，我们会提供给你双眼的治疗方案。

**隐私的保密**

作为研究的一部分，研究医生和研究人员将收集你的医学和个人信息用于研究和科学分析，你的信息将会以书面和电子储存形式保存。为检查是否正确的进行了研究，为负责审批的监管机构、伦理委员会、研究人员的代表，都能接触到你的信息。签署了这份知情同意书，就表明你和你的监护人允许有合法理由的人员查看你的相关信息。

你与研究医生协商后可以查看所收集到的关于你的信息，并可以要求根据实际情况进行修改。任何时候你都可以退出研究，但在法律允许的范围内，你退出前收集的信息将会继续被有合法理由的人员使用。

为了保护隐私，研究医生将会对你的某些信息以编码形式传送，这些信息不包括姓名、地址或其他可以直接辨认你身份的标志。这些信息上有一个编码，只有你的研究医生能够将这个编码与你的姓名联系起来。在科学会议或者科学杂志上发表本研究获得的研究信息和数据时，你的身份也不会被公开。

**联系方式**

如果你和你的监护人有与本研究相关的任何问题，或有任何紧急情况请联系＜ ＞医生，工作时间请联系 ，下班时间、周末和节假日联系 。

如果你和你的监护人有与自身权利/权益相关的任何问题，或者想反映参与本研究过程中遭遇的困难、不满和忧虑，或者想提供与本研究有关的意见和建议，请联系 伦理委员会办公室电话： ，电子邮件： 。

知情同意书签字页

-----------------------------------------------------------------------------

签署此页表明我已了解下列事项：

- 我已经阅读并理解了知情同意书的所有内容，而且已经充分考虑。
- 我的所有疑问己得到满意答复。
- 我同意自愿加入此项研究，将遵守研究流程，且将在需要时向研究者或工作人员提供必要的信息。
- 我理解可以在研究的任何时候自由退出此项研究。
- 我将得到此份知情同意书的复印件。

**受试者同意声明：**我已被充分告知了有关这项试验的上述情况，所提出的所有问题已得到了满意的回答。我自愿参加这项试验，并愿意在试验中与医生充分合作完成所规定的诊疗及相关检查。

未成年受试者签名：____________________________  日期：______________

未成年受试者姓名正楷：____________________________________________

法定监护人签名（必须）：____________________ 日期：_____________

法定监护人姓名正楷：____________________ ______________________

需法定代理人签署的原因及关系：**__________________________________________**

见证人（如适用）签名：________________________ 日期： _____________

见证人（如适用）姓名正楷：________________________________________

需见证人签署的原因：________________________________________

**研究者声明：**我确认已向受试者及其监护人详尽解释了本临床试验的有关内容，包括受试者可能的获益和风险，并给其一份签署过的知情同意书复印件。

研究者签名： 签署日期：

研究者正楷姓名：
